# Vanillic Acid Improves Comorbidity of Cancer and Obesity through STAT3 Regulation in High-Fat-Diet-Induced Obese and B16BL6 Melanoma-Injected Mice

**DOI:** 10.3390/biom10081098

**Published:** 2020-07-24

**Authors:** Jinbong Park, Seon Yeon Cho, JongWook Kang, Woo Yong Park, Sujin Lee, Yunu Jung, Min-Woo Kang, Hyun Jeong Kwak, Jae-Young Um

**Affiliations:** 1 Department of Pharmacology, College of Korean Medicine, Kyung Hee University, Seoul 02447, Korea; thejinbong@khu.ac.kr (J.P.); darkzney@daum.net (J.K.); jwy87@naver.com (Y.J.); 2 Department of Comorbodity Research, KyungHee Institute of Convergence Korean Medicine, Kyung Hee University, Seoul 02447, Korea; 3 Department of Science in Korean Medicine, Graduate School, Kyung Hee University, 26 Kyungheedae-ro, Dongdaemun-Gu, Seoul 02447, Korea; choseonyeon@naver.com (S.Y.C.); jjing0429@naver.com (W.Y.P.); cjfrkqkd10@naver.com (S.L.); 4 Department of Korean Medicine, Graduate School, Kyung Hee University, 26 Kyungheedae-ro, Dongdaemun-Gu, Seoul 02447, Korea; dokangsss@hanmail.net; 5 Department of Natural Science, College of Convergence and Integrated Science, Kyonggi University, Suwon 16227, Korea; hjkwak@kyonggi.ac.kr

**Keywords:** vanillic acid, STAT3, cancer, obesity, comorbidity, melanoma, autophagy

## Abstract

Obesity is known to be associated with risk and aggressiveness of cancer. Melanoma, the most lethal type of skin cancer, is also closely related to the prevalence of obesity. In this study, we established a cancer–obesity comorbidity (COC) model to investigate the effects of vanillic acid (VA). After a five-week administration with a high-fat diet (HFD) to induce obesity, subcutaneous allograft of B16BL6 cells were followed, and VA was orally administrated for an additional two weeks. VA-fed mice showed significantly decreased body weight and white adipose tissue (WAT) weight, which were due to increased thermogenesis and AMPK activation in WATs. Growth of cancer was also suppressed. Mechanistic studies revealed increased apoptosis and autophagy markers by VA; however, caspase 3 was not involved. Since signal transducer and activator of transcription 3 (STAT3) is suggested as an important pathway linking obesity and cancer, we further investigated to find out if STAT3 phosphorylation was repressed by VA treatment, and this was again confirmed in a COC cell model of adipocyte conditioned medium-treated B16BL6 melanoma cells. Overall, our results show VA induces STAT3-mediated autophagy to inhibit cancer growth and thermogenesis to ameliorate obesity in COC. Based on these findings, we suggest VA as a candidate therapeutic agent for COC treatment.

## 1. Introduction

Obesity is one of the leading causes of chronic diseases and morbidity, including cancer, in which obesity accounts for almost 20% of all cases [1]. Although the mechanistic link is yet to be clarified, the close relevance between obesity and cancer is now a well-accepted theory. Melanoma, the most serious type of skin cancer, also falls in line. Various studies have discovered an epidemiological relevance between excess adiposity and malignant melanoma [2,3,4]. Moreover, according to a meta-analysis of 10 cohort (7895 cases in 6,368,671 subjects) and 11 case-control studies (4460 cases and 6342 controls), a positive association between obesity and melanoma in men was reported [5].

Numerous efforts have been made to elucidate the underlying connection between obesity and cancer. Our previous work regarding adipocytes and melanoma revealed the fact that adipocyte-conditioned medium induced proliferation and migration of B16BL6 melanoma cells via Akt/mTOR signaling [6]. Jung et al. suggest the role of inflammation, especially M2 macrophages, within the cancer microenvironment [7]. An experimental approach by Malvi et al. showed that Orlistat, which is a widely used anti-obesity agent, reversed high-fat diet (HFD)-induced melanoma progression, suggesting the close link between obesity and melanoma malignance depends on adipokines [8]. Another possible factor is exosomes derived from adipocytes. In 2016, Lazar and colleagues suggested that exosomes derived from adipocytes induce melanoma aggressiveness by increasing fatty acid oxidation [9].

An important cancer survival pathway activated in obesity is signal transducer and activator of transcription 3 (STAT3). STAT3, a member of the STAT protein family, is phosphorylated in response to cytokines and growth factors (GFs) and enters the nucleus to act as a transcription factor [10]. Though STAT3 is essential for survival during early embryogenesis [11], increased activation of STAT3 is often found in cancer cells, which, in turn, drives the downstream protein complexes to prolong survival [12]. STAT3 is also a possible factor which links obesity and cancer. In particular, interleukin 6 (IL-6) and leptin, which are closely related to the mass of white adipose tissue (WAT) [13], are known to activate STAT3 in cancer cells or tissues [14,15]. The downstream target of mitogen-activated protein kinases (MAPKs) can also activate STAT3. Activated STAT3, in turn, regulates transcription pathways, which suppress apoptosis and promote the survival of cancer cells [16]. However, the exact molecular mechanisms which link obesity to aggressiveness of melanoma remain as a mystery.

Vanillic acid (VA, 4-hydroxy-3-methoxybenzoic acid) is an aromatic acid derived from numerous plant sources such as *Euterpe oleracea* (Acai berry) [17], *Angelica sinensis* [18], and *Panax ginseng* [19]. VA is well-reported to exert anticancer activities in several types of cancers, including lymphocytes [20], leukemia [21], colon cancer [22], lung cancer [23], and also melanoma [19]. Moreover, we showed its anti-obesity effect in our previous study [24]. Since references support the potentially beneficial properties of VA in a comorbid situation of cancer and obesity, under this hypothesis, we establish a cancer–obesity comorbidity model (COC) of melanoma based on a study by Jung et al. [7] and investigate the effect of VA focusing on the role of STAT3 pathway.

## 2. Materials and Methods

### 2.1. Chemical Reagents

VA was purchased from Sigma Chemicals Co. (St. Louis, MO, USA). VA was dissolved in dimethyl sulfoxide (DMSO). Dulbecco modified eagle medium (DMEM) medium and fetal bovine serum (FBS) were obtained from Gibco (Grand Island, NY, USA). Insulin, 3-isobutylmethylxanthine (IBMX), and dexamethasone were obtained from Sigma-Aldrich (St Louis, MO, USA). Poly-vinylidene difluoride (PVDF) was procured from Millipore (Merck KGaA, Darmstadt, Germany). The electrochemiluminescence (ECL) kit was obtained from GE Healthcare Life Sciences (Seoul, Korea).

### 2.2. Antibodies

Anti-vascular endothelial growth factor (VEGF), anti-vimentin, anti-nuclear factor kappa-light-chain-enhancer of activated B cells (NFkB) and anti-cyclooxygenase-2 (COX-2), muscle RING-finger protein 1 (Murf1), and muscle atrophy F-Box (MAFbx) antibodies were purchased from Santa Cruz Biotechnology (Dallas, TX, United States). Antibodies for peroxisome proliferator-activated receptor gamma coactivator 1-alpha (PGC1α), PR domain zinc-finger protein 16 (PRDM16), hormone sensitive lipase (HSL), adipose tissue triacylglycerol (ATGL), and 1-acylglycerol-3-phosphate O-acyltransferase ABHD5 (CGI58) were purchased from Abcam (Cambridge, United Kingdom). Anti-uncoupling protein 1 (UCP1) antibody was purchased from GeneTex (Irvine, CA, United States) and antibodies for phospho-HSL, β-actin, mouse double minute 2 homolog (MDM2), phospho-Unc-51 like autophagy activating kinase (ULK) 1 (S555, S757), Beclin-1, peroxisome proliferator-activated receptor gamma (PPARγ), phospho-AMP-activated protein kinase (AMPK), AMPK, phospho-acetly-CoA carboxylase (ACC), ACC, phospho-p53, BAX, Caspase3, autophagy related 7 (ATG7), autophagy related 12 (ATG12), microtubule-associated protein 1A/1B-light chain 3 (LC3), phospho-c-jun N-terminal kinase (JNK), JNK, phospho-p38, p38, phospho-extracellular-signal-regulated kinase (ERK), ERK, phospho-mitogen-activated protein kinase/ERK kinase (MEK) 1/2 (S217/221), phospho-STAT3, STAT3, and suppressor of cytokine signaling 3 (SOCS3) were purchased from Cell Signaling Technology (Beverly, MA, United States). Anti-Bcl-2 antibody was purchased from Thermo Fisher Scientific (Waltham, MA, United States), and anti-CD137 antibody was purchased from the Institute of Systems Biotechnology (iSBio, Saar-Uni, Germany).

### 2.3. Animal Experiment

Male C57BL6/J mice (6 weeks old) were purchased from Daehan Biolink Co. (Eumsung, South Korea). Mice were maintained on a 12 h light/dark cycle in a pathogen-free animal facility and provided with diet and water ad libitum for 1 week prior to the experiments. To induce obesity, mice were fed an HFD (Rodent Diet D12492; Research Diet, New Brunswick, NJ, United States) consisting of 60% fat for 5 weeks. After we induced obesity, the mice (*n* = 15) were randomly divided into three groups, and two of three groups were subcutaneously injected with 1 × 10^4^ cells of B16BL6 melanoma in the right leg. Two days after tumor inoculation, VA (10 mg/kg/day) was administered via oral gavage every other day, for two weeks, to one of the tumor-injected groups. The dose of VA was determined based on our previous study [24]. All groups were maintained with HFD administration. After the experiment, the mice were sacrificed under CO_2_ anesthesia, and inguinal white adipose tissue (iWAT), epididymal white adipose tissue (eWAT), brown adipose tissue (BAT), gastrocnemius muscle, tibialis anterior muscle, liver, and spleen tissues were collected for further analysis. All animal experiments were performed in accordance with the ethical guidelines of Kyung Hee University and approved by the Institutional Review Board of Kyung Hee University (confirmation number: KHUASP (SE)-15-08).

### 2.4. Protein Extraction and Western Blot Analysis

Cells and tissues were lysed for 30 min on ice through radioimmunoprecipitation assay (RIPA) buffer (Cell Signaling Technology, Danvers, MA, USA). Thereafter, insoluble solutes were removed by centrifugation at 13,000 rpm for 20 min at 4 °C. The lysates were quantified by DC protein assay and then sampled (Bio-Rad, Hercules, CA, USA). The lysates were resolved by sodium dodecyl sulfate (SDS)-polyacrylamide gel electrophoresis and transferred onto a PVDF membrane. Membranes were blocked with 5% skim milk for 1 h, and the primary antibody (1:1000) was incubated overnight at 4 °C. The next day, horseradish peroxidase (HRP)-conjugated secondary antibody (1:5000) was incubated at room temperature for 1 h. The protein signals were detected by using the ECL advance kit. Blot expressions from separate Western blot experiments were normalized after setting vehicle-treated COC mice as 100%, to confirm relative changes between groups.

### 2.5. Hematoxylin and Eosin (H&E) Staining

H&E staining was performed as previously described [25]. In brief, WATs were washed in PBS and fixed in 10% formalin for 2 weeks. Then, the tissues were embedded in paraffin. The tissue sections were deparaffinized in xylene and rehydrated with ethanol/water and then stained with hematoxylin–eosin (H&E). Microscopic examinations were performed, and photographs were taken under a regular light microscope. The average adipose droplet size was calculated by using the Image J software program (National Institute of Health, Bethesda, MD, USA).

### 2.6. Adipocyte Differentiation and Conditioned Media (CM) Harvest

We performed 3T3-L1 adipocyte culture and differentiation as previously described [26]. Briefly, 3T3-L1 preadipocytes were grown in DMEM containing 10% BS and 100 units/mL of P/S/G solution in 5% CO_2_, at 37 °C, until cells were fully confluent. After 2 days from full confluence (Day 0), the cells were differentiated by a 48 h incubation in differentiation medium consisting of DMEM plus 10% FBS containing 0.5 mM IBMX, 1 mM dexamethasone, and 1 mg/mL insulin. On Day 2, the cells were cultured in DMEM plus 10% FBS supplemented with 1 mg/mL for another 48 h, followed by fresh DMEM culture medium containing 10% FBS and 1 mg/mL insulin. Fully differentiated cells were replaced with DMEM containing 1% FBS, and the culture medium was harvested/replaced every 24 h for a total of 3 days. Collected medium was centrifuged at 2000 rpm for 3 min, to separate the supernatant, and the collected supernatant medium was filtered through a 22 μm sterile syringe filter (CORNING, New York, NY, USA). The media were mixed in equal proportions and diluted in DMEM containing 10% FBS, for further experiments.

### 2.7. Cell Growth Assay

B16BL6 melanoma was seeded (1 × 10^3^ cells per well) in 96-well plates and treated with various concentrations (0–100%) of CM. Cell growth rate was measured for every 24 h, for 4 days, using a cell proliferation MTS kit from Promega Corporation (Madison, WI, USA). The absorbance was measured at 450 nm in a VERSAmax microplate reader (Molecular Devices, Sunnyvale, CA, USA) to determine the formazan concentration, which is proportional to the number of live cells.

### 2.8. Cytotoxicity Assay

B16BL6 melanoma was seeded (2 × 10^4^ cells per well) in 96-well plates and treated with various concentrations (0.01–20 µM) of VA for 24 h. Cell viability was measured by using the cell proliferation MTS Kit from the Promega Corporation (Madison, WI, USA).

### 2.9. Immunofluorescence Staining

The cells were fixed by using 10% formalin and blocked with 5% BSA for 1 h. After, the cells and tissue were incubated with the indicated primary antibodies (anti-p-STAT3 and anti-β-actin, 1:50 in 5% BSA), overnight, at 4 °C. After their washing, the cells and tissue were incubated with Alexa Flour 488 or 633-conjugated secondary antibody (1:1000), and the fluorescence was detected by using an EVOSR Cell Imaging system (Thermo Scientific, Carlsbad, CA, USA). DAPI was used to stain cell nuclei.

### 2.10. Statistical Analysis

Data were expressed as mean ± standard error mean (SEM) of three or more independent experiments. Statistical differences were calculated by one-way ANOVA and a subsequent post hoc Tukey test, unless stated otherwise. All statistical analysis was completed by using SPSS statistical analysis software version 11.5 (SPPS Inc., Chicago, IL, USA). Probability values of *p* < 0.05 were used as the criterion for statistical significance.

## 3. Results

### 3.1. VA Ameliorated Obesity-Related Parameters in a COC Mouse Model

**Figure 1:**
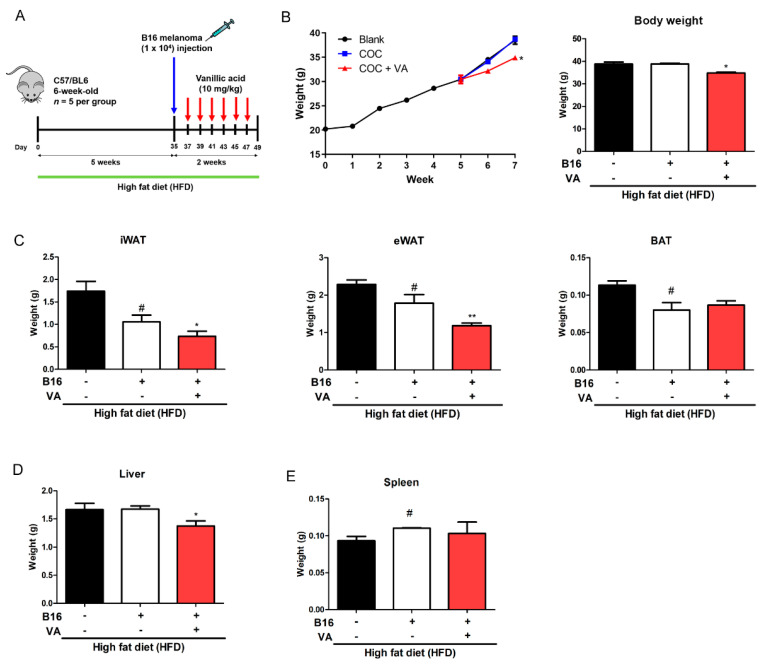
Effect of VA on obesity parameters in COC mice. (**A**) Experimental scheme of in vivo study. After HFD feeding of five weeks, mice (*n* = 15) were randomly divided into three groups. Then, 1 × 10^4^ B16BL6 melanoma cells were injected subcutaneously into the right leg in two groups. Two days after tumor inoculation, VA (10 mg/kg/day) was administered orally for two weeks to one of the melanoma-injected groups. (**B**) Weekly change in body weight and final body weight were measured. (**C**) Adipose tissue (iWAT, eWAT, and BAT), (**D**) liver tissue, and (**E**) spleen tissue weight was measured. All data are expressed as mean ± SEM (*n* = 5); ^#^ *p* < 0.05 vs. HFD-fed control mice; * *p* < 0.05 and ** *p* < 0.01 vs. vehicle-treated COC mice. COC, cancer–obesity comorbidity; HFD, high-fat diet; VA, vanillic acid; iWAT, inguinal white adipose tissue; eWAT, epididymal white adipose tissue; BAT, brown adipose tissue; GAS, gastrocnemius muscle; TA, tibialis anterior muscle.

First, to establish a COC model in mice, we fed C57BL6 mice a HFD for five weeks and injected B16BL6 melanoma cells (1 × 10^4^ cells) subcutaneously into the right flank. The test group (VA group) received an oral administration of VA at 10 mg/kg/day, and the comorbidity group (COC group) received an equivalent amount of DMSO. The normal control group (Blank group) was not injected with melanoma cells. All groups were fed with HFD for additional 2 weeks (Figure 1A). As shown in Figure 1B, the body weight of melanoma-injected COC group did not differ from control group; however, VA-fed mice showed decreased body weight, around 4 g. The weight of tissues was also affected by VA treatment. Cancer injection resulted in decreased weight in iWAT, eWAT, and BAT, of which weights were further repressed by VA treatment, except with BAT (Figure 1C). While liver tissue weight was not changed in COC mice, VA-treated mice showed significantly decreased liver weight (Figure 1D). The decrease in VA-treated mice was due to alteration of lipolysis when confirmed by Western blot analysis of lipolysis markers, including HSL and ATGL (Supplementary Materials Figure S1). Spleen weight was not different in all groups (Figure 1E). Based on these results, we could conclude VA suppresses HFD-induced body weight gain by reducing fat accumulation without any negative impact on other organs.

### 3.2. VA Did Not Drive Cachectic Actions in Muscle Tissue of COC Mice

**Figure 2:**
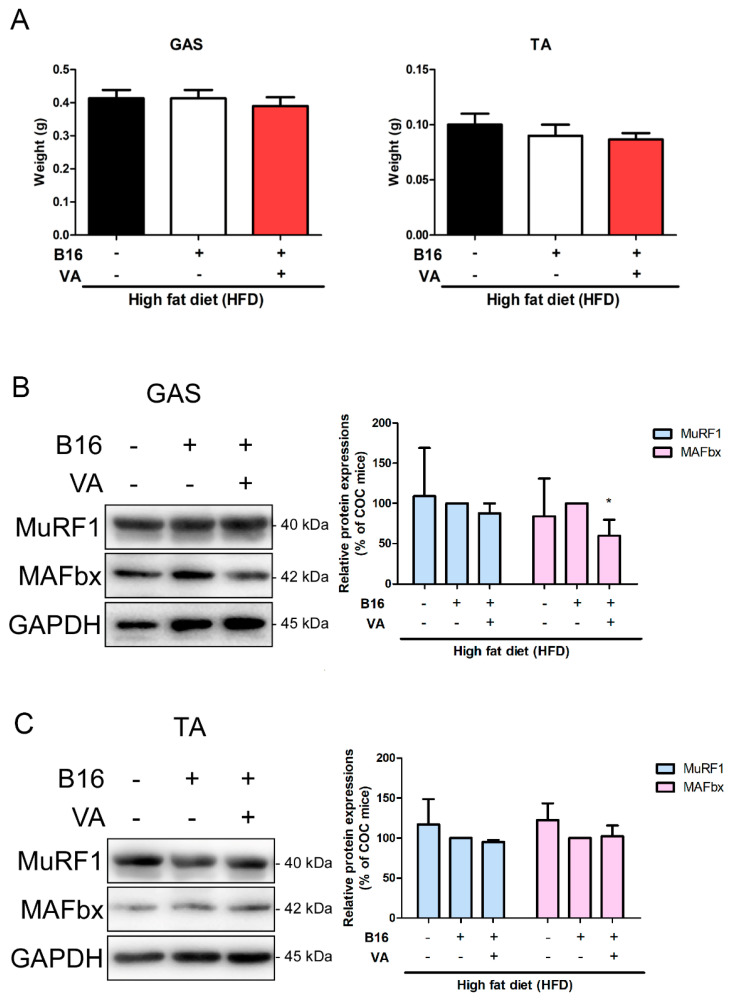
Effect of VA on cachexia parameters in COC mice. (**A**) Muscle tissue weights of gastrocnemius and tibialis anterior were measured. Protein levels of (**B**) MuRF1 and (**C**) MAFbx were analyzed by a Western blot analysis. Results were expressed relative to β-actin. All data are expressed as mean ± SEM (*n* = 5); * *p* < 0.05 vs. vehicle-treated COC mice. COC, cancer–obesity comorbidity; HFD, high-fat diet; VA, vanillic acid; GAS, gastrocnemius muscle; TA, tibialis anterior muscle.

Since VA treatment reduced body weight and adipose tissue weight, we confirmed whether cachectic events were occurred. As shown in Figure 2A, tissue weight in two different muscles, gastrocnemius and tibialis anterior, did not differ from the non-cancer group or vehicle-treated COC group. The main mechanism of pathologic muscle decrease during cancer-associated cachexia is muscle atrophy caused by abnormal proteolysis [27]. When muscle proteolysis markers, including MuRF1 and MAFbx, were assessed by Western blot analysis, we could observe no significant alteration of these proteins in either of the muscle tissues (Figure 2B,C).

### 3.3. VA Induced Browning and Lipolysis in iWAT of COC Mice

**Figure 3:**
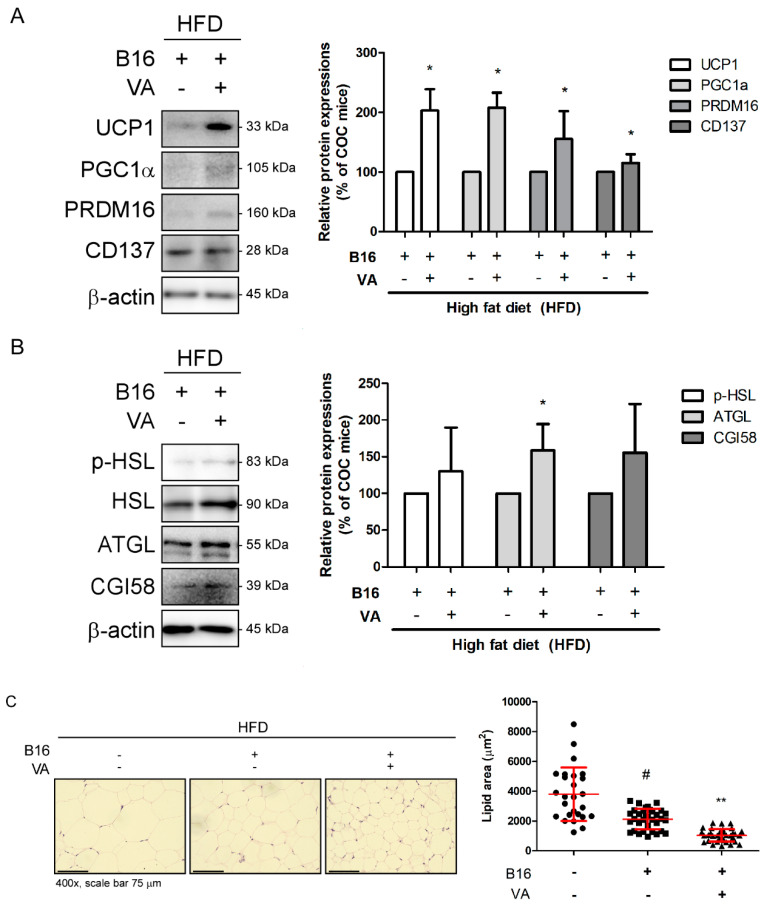
Effect of VA on iWAT of COC mice. (**A**) Protein levels of UCP1, PGC1α, PRDM16, and CD137 were analyzed by a Western blot analysis. Results were expressed relative to β-actin. (**B**) Protein levels of p-HSL, ATGL, and CGI58 were analyzed by a Western blot analysis. Results were expressed relative to β-actin, except p-HSL, which was normalized to total HSL. (**C**) H&E staining was performed in paraffin-embedded iWAT (magnification ×400, scale bar 75 μm), and average lipid droplet size was measured. All data are expressed as mean ± SEM (*n* = 3); ^#^ *p* < 0.05 vs. HFD-fed control mice; * *p* < 0.05 and ** *p* < 0.01 vs. vehicle-treated COC mice. COC, cancer–obesity comorbidity; HFD, high fat diet; VA, vanillic acid; iWAT, inguinal white adipose tissue.

Our previous study showed that VA can ameliorate obesity by inducing non-shivering thermogenesis in WAT [24]. Therefore, to verify the mechanism of the WAT-reducing effect of VA, we performed Western blot assays in iWAT of COC and VA-fed COC mice. As in Figure 3A, we could observe highly altered levels of UCP1, PCG1α, and PRDM16, which are all factors related to non-shivering thermogenesis [28]. Moreover, CD137, a beige specific marker [28], was also increased, suggesting that VA induced browning in iWAT. Next, to assess whether VA treatment upregulated lipid metabolism as well, we evaluated expression levels of lipolysis markers, such as HSL, ATGL, and CGI58 [29]. Although all of these factors showed a tendency to increase by VA, only ATGL showed statistical significance (Figure 3B). Consistent to the results indicating VA-induced thermogenic and lipolytic alteration, H&E staining clearly revealed the decrease in size of lipid droplets (Figure 3C).

### 3.4. VA Activated AMPK Pathway and Increased UCP1 Expression in eWAT of COC Mice

**Figure 4:**
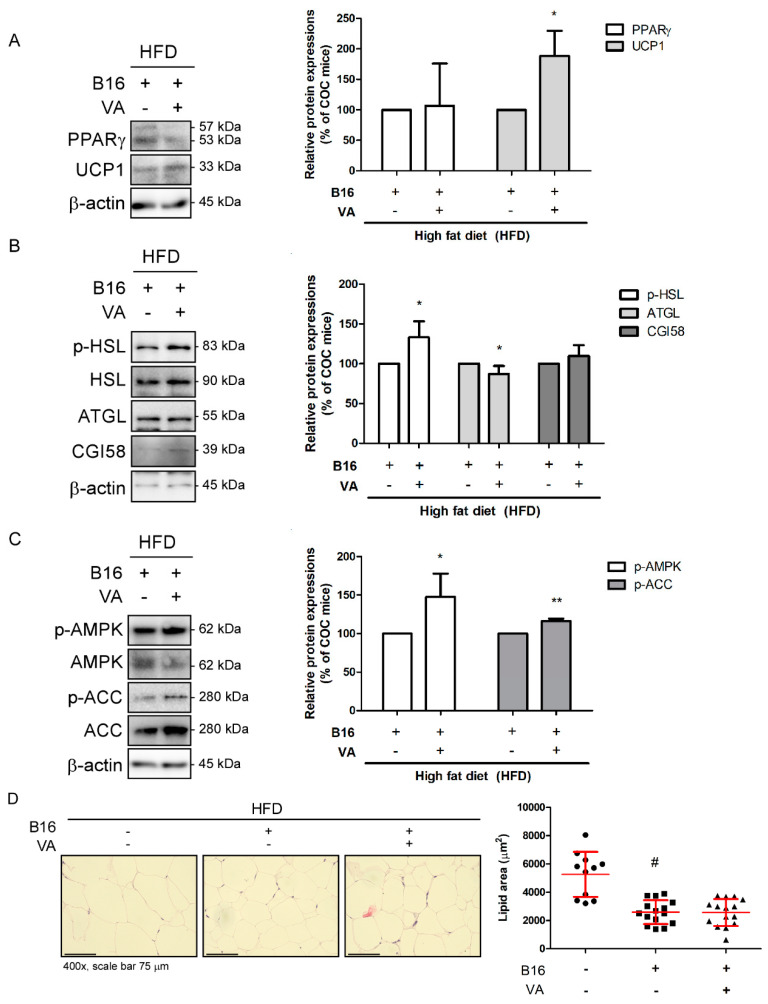
Effect of VA on eWAT of COC mice. (**A**) Protein levels of PPARγ and UCP1 were analyzed by a Western blot analysis. Results were expressed relative to β-actin. (**B**) Protein levels of p-HSL, ATGL, and CGI58 were analyzed by a Western blot analysis. Results were expressed relative to β-actin, except p-HSL, which was normalized to total HSL. (**C**) Protein levels of p-AMPK and p-ACC were analyzed by a Western blot analysis. β-actin was used as a loading control. Results of p-AMPK and p-ACC were normalized to total AMPK and ACC, respectively. (**D**) H&E staining was performed in paraffin-embedded eWAT (magnification ×400, scale bar 75 μm), and average lipid droplet size was measured. All data are expressed as mean ± SEM (*n* = 3); ^#^ *p* < 0.05 vs. HFD-fed control mice; * *p* < 0.05 and ** *p* < 0.01 vs. vehicle-treated COC mice. COC, cancer–obesity comorbidity; HFD, high-fat diet; VA, vanillic acid; eWAT, epididymal white adipose tissue.

Since eWAT weight was also decreased by VA treatment, we next evaluated changes in eWAT. As shown in Figure 4A, compared to COC mice, VA treatment did not alter the expression of PPARγ; however, UCP1 protein expression was significantly increased. When we investigated changes in lipolysis markers, controversial results were observed. VA treatment induced phosphorylation of HSL, while slightly suppressing ATGL. The expression of CGI58 remained unchanged (Figure 4B). Our previous work regarding VA revealed its effect on AMPK activation in an obesity mouse model [24], suggesting the energy metabolism regulator AMPK [30] may play a role in the current model of COC, as well. Supporting such evidence, further Western blot results confirmed phosphorylation of AMPK, and its downstream target ACC was induced in VA-treated COC mice (Figure 4C). In consistent to histological analysis in iWAT, VA also decreased the size of lipid droplets within eWAT, as well (Figure 4D).

### 3.5. VA Reduced Tumor Growth and Induced STAT3 Activation and Autophagy in Melanoma Tissue of COC Mice

**Figure 5:**
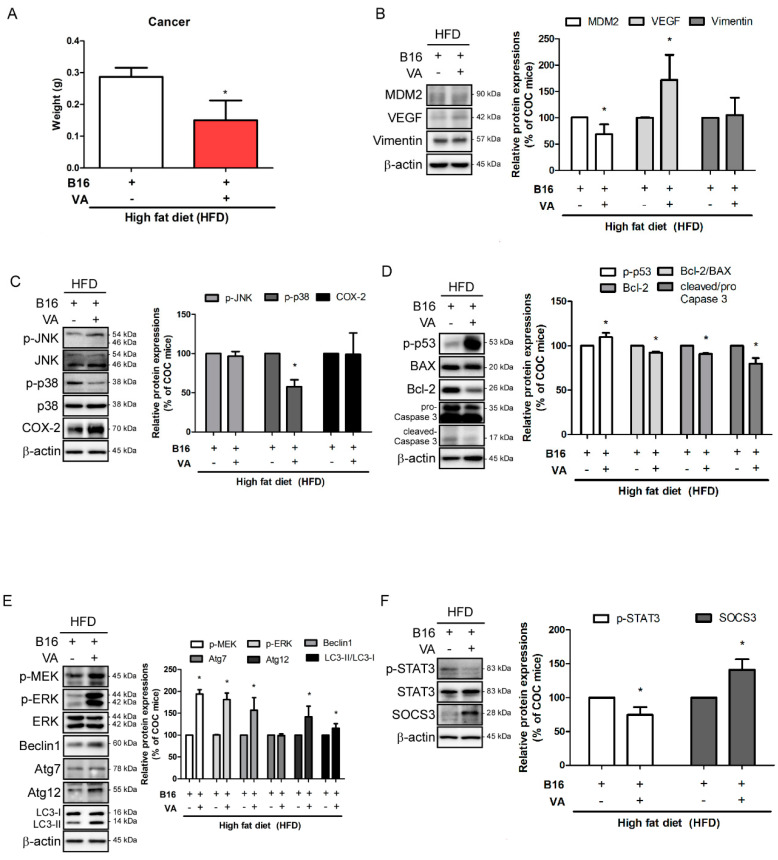
Effect of VA on allografted B16BL6 melanoma of COC mice. (**A**) Final weight of allografted B16BL6 melanoma tissue was measured. (**B**) Protein levels of MDM2, VEGF, and Vimentin were analyzed by a Western blot analysis. Results were expressed relative to β-actin. (**C**) Protein levels of p-JNK, p-p38, and COX-2 were analyzed by a Western blot analysis. Results of p-JNK and p-p38 were normalized to expressions of total JNK and p38, respectively. Result of COX-2 was expressed relative to β-actin. (**D**) Protein levels of p-p53, Bcl-2, BAX, and Caspase 3 were analyzed by a Western blot analysis. Results were expressed relative to β-actin. (**E**) Protein levels of p-MEK, p-ERK, Beclin-1, Atg7, Atg12, and LC3-I/II were analyzed by a Western blot analysis. Results were expressed relative to β-actin, except p-ERK, which was normalized total ERK. (**F**) Protein levels of p-STAT3 and SOCS3 were analyzed by a Western blot analysis. Results of p-STAT3 and SOCS3 were expressed relative to total STAT3 and β-actin, respectively. All data are expressed as mean ± SEM (*n* = 3); * *p* < 0.05 vs. vehicle-treated COC mice. COC, cancer–obesity comorbidity; HFD, high-fat diet; VA, vanillic acid; eWAT, epididymal white adipose tissue.

In our COC mouse model, VA treatment significantly reduced the tissue weight of allografted melanoma (Figure 5A). Thus, we investigated the underlying action mechanisms of VA which affected the size of cancer. First, we evaluated markers of proliferation and survival of melanoma. Western blot analysis in factors which participate in angiogenesis of cancer showed controversial results. While expression of Vimentin remained unchanged, MDM2 was decreased and VEGF was increased by VA treatment (Figure 5B). MAPKs, markers of inflammation which also drive proliferation of cancer cells [31], were not affected much, as JNK activation was unaffected and only phosphorylation of p38 was decreased in cancer tissues of VA-treated mice. COX-2, an enzyme and also an important indicator of inflammation [32], also showed no difference between COC mice and VA-treated COC mice (Figure 5C).

One of the most important therapeutic targets for cancer regulation is apoptosis. Therefore, we further verified whether VA altered apoptosis in the cancer tissue. As shown Figure 5D, VA induced phosphorylation of the tumor-suppressor protein p53 [33], while apoptotic markers Bcl2/β-actin and Bcl2/BAX ratio [34] were decreased, as well. However, cleavage of caspase 3 was unexpectedly decreased, suggesting that the cytotoxic effect of VA in melanoma is independent from caspase 3-mediated apoptosis. In addition to apoptosis, autophagy is another type of programmed cell death which is considered an attractive target pathway for cancer treatment. The results clearly indicated altered autophagy in melanoma tissues of VA-treated mice. VA increased phosphorylation of MEK and ERK, which participate in the later step of autophagy [35], along with increased autophagy markers, such as Beclin-1, ATG7, and ATG12, and conversion of LC3-I to LC3-II [36] (Figure 5E).

Studies suggest STAT3 as a regulator for both apoptosis- and autophagy-mediated cell death [37,38]. Our results support such a notion, since decreased melanoma weight by treating VA was a result of regulated STAT3. As shown in Figure 5F, VA-treated mice significantly reduced phosphorylation of STAT3 while inducing SOCS3 expression. Overall, the in vivo experiment suggested that a VA-induced decrease of melanoma weight is closely related to STAT3 activation and consequent regulation of apoptosis and autophagy.

### 3.6. VA Inhibited Phosphorylation of STAT3 in B16BL6 Melanoma Cells Treated with Adipocyte CM

**Figure 6:**
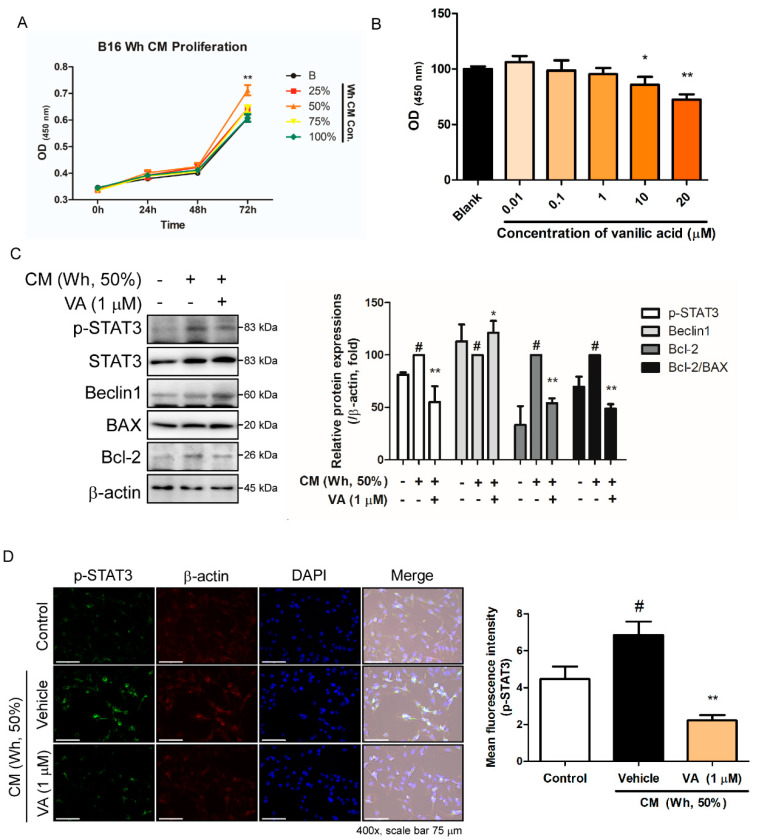
Effect of VA on adipocyte CM-treated B16BL6 melanoma cells. (**A**) The cell growth rate of B16BL6 melanoma according to the concentration (0%, 25%, 50%, 75%, and 100%) of CM diluted in complete medium was measured by an MTS assay. (**B**) Cytotoxicity of VA in B16BL6 melanoma was determined by an MTS assay. (**C**) Protein levels of p-STAT3, Beclin-1, BAX, and Bcl-2 were analyzed by a Western blot analysis. Results were expressed relative to β-actin, except p-STAT3, which was normalized to total STAT3. (**D**) Expression of intracellular p-STAT3 was evaluated by an immunofluorescence staining (magnification ×400, scale bar 75 μm). All data are expressed as mean ± SEM (*n* = 3); ^#^ *p* < 0.05 vs. untreated B16BL6 melanoma cells; * *p* < 0.05 and ^**^ *p* < 0.01 vs. CM-treated B16BL6 melanoma cells. CM (Wh), white adipocyte conditioned media; VA, vanillic acid.

To confirm the in vivo results, we conducted further in vitro studies by mimicking the adipose tissue effect in melanoma cells. By treating B16BL6 melanoma cells with CM from mature 3T3-L1 adipocytes for 72 h, we observed altered proliferation of melanoma cells, especially when the ratio of CM and fresh culture medium was 1:1 (Figure 6A). Thus, 50% CM with fresh DMEM and 10% FBS was selected as the proper condition for COC cell model. Next, to verify treatment concentration of VA, we evaluated cytotoxicity of VA in B16BL6 melanoma cells, to find out 0.01–1 μM of VA does not affect cell viability (Figure 6B). Since we aimed to assess the effect of VA on COC instead of direct effect on cancer cell death, we further investigated the effect of 1 μM of VA in 50% CM-treated B16BL6 melanoma cells. Western blot analysis on related markers indicated that CM treatment indeed repressed the autophagic marker Beclin-1 and apoptosis marker Bcl2/BAX. However, VA treatment restored these markers, suggesting that VA drives apoptosis and autophagy via suppression of STAT3 phosphorylation in the COC cell model (Figure 6C). By additional IF staining, we could confirm VA treatment significantly suppressed phosphorylation of STAT3 (Figure 6D), consistent to the in vivo results from COC mice.

## 4. Discussion

Epidemiological studies support the notion that obesity exacerbates cancer. Statistically, obesity accounts for 20% of all cancer cases [1]. Overweight and obesity are also associated with increased risk of malignant melanoma [5]. However, the exact interplay in which obesity and adipose tissue worsen cancer malignancy is not clearly understood. One promising pathway explaining the relevance between obesity and cancer is the STAT3 signaling pathway [16]. STAT3, a member of the STAT family, participates in numerous physiological signaling pathways [39], including a critical role during early embryonic development [11]. Regarding the fact STAT3 is involved in numerous processes, its importance has been particularly accepted in the development of cancer. Since increased activation of STAT3 is observed in a wide variety of cancer cells, and it regulates downstream channels to promote survival and growth, researchers have pointed out STAT3 as a promising molecular target for therapeutic intervention in cancer patients. In this study, we attempted to verify the effect of the naturally occurring aromatic acid VA on COC by investigating its specific action in STAT3 activity. We demonstrated a beneficial effect of VA in a COC model of HFD-fed obese mice injected with B16BL6 melanoma cells. The results showed that VA treatment significantly reduced the size of cancer whilst ameliorating obesity parameters. This clearly indicates VA could benefit COC by improving symptoms of both obesity and cancer (Figure 1).

Adipogenesis is a complicated process in which various factors form a highly coordinated network. STAT3 pathway is activated during the early steps of adipogenesis [40,41], and later studies found out that STAT3 activity is necessary in adipogenesis by reporting interrupted STAT3 activation or synthesis leading to impaired adipocyte development [42,43]. In addition to its role during adipogenesis, mature adipocytes also secrete cytokines (cardiotrophin-1, IL-6) and hormones (leptin, prolactin) that engage the STAT3 signaling pathway in target tissues [44]. Cancer tissues are not an exception. The excessive STAT3 activation in cancer cells is closely connected to the surrounding cancer microenvironment, as well. By adipocyte-derived molecules, STAT3 activity in cancer is also upregulated, leading to a significant drive in the survival mechanism. STAT3 is considered as an important oncogene and as an effective therapeutic target for cancer treatment. It is widely accepted that numerous cytokines, GFs, and other oncogenes can activate STAT3 canonically, and thus constitutive activity of STAT3 is observed in high ratio of cancer [45]. In addition to its role within the nucleus to potentiate survival, proliferation, and metastasis through transcriptional activity, cytoplasmic STAT3 also affects cancer cells by controlling metabolism, immune evasion, angiogenesis, and programmed cell death via non-transcriptional regulation [46]. Our results from VA-treated COC mice showed a significant reduction in phosphorylation of STAT3 by VA administration, suggesting its beneficial activity upon STAT3 regulation (Figure 5F). Increase of STAT3 phosphorylation associated with proliferation was also reproduced in the in vitro model of which we treated B16BL6 melanoma cells with CM from mature adipocytes (Figure 6). In this COC cell model, VA treatment successfully repressed STAT3 activation, in turn inducing the apoptotic and autophagic pathway in melanoma cells.

In melanoma, the ratio of BAX, a pro-apoptotic protein, and Bcl-2, an anti-apoptotic protein, is considered to be important in relation to the susceptibility of melanoma to apoptosis [47]; thus, these proteins are considered as a therapeutic target [48,49]. In addition to these Bcl-2 family proteins, persistently altered STAT3 activity is known to promote the proliferation and survival of cancer cells via regulation of apoptosis [50]. Moreover, STAT3 can also suppress transcription of the well-established tumor suppressor p53 [51]. Investigation on melanoma samples revealed constitutive STAT3 activation is associated with anti-apoptotic factors such as Bcl-xL upon consistent progression [52]. In line with this, Wu et al. [53] reported that STAT3 inhibition induced apoptosis specifically in malignant melanoma cell line A375, but not in other types of cancers. These reports suggest that the regulation of STAT3 activity is a potential therapeutic target for melanoma treatment. In accordance with the inhibited phosphorylation of STAT3 by VA, our results showed that VA treatment increased pro-apoptotic markers such as phosphorylation of p53 and Bcl-2/BAX ratio, but on the other hand, the final regulator of apoptosis, caspase 3, was not activated but rather slightly decreased. However, a significant decrease in melanoma tissue associated with an unexpected decrease in cleavage of caspase 3 suggested VA-induced melanoma cell death via a different pathway besides apoptosis (Figure 5D).

In addition to apoptosis, autophagy is also a well-established form of programmed cell death. The crosstalk between autophagy and STAT3 signaling may decide the survival or death of a cell. Several studies imply the participation or contribution of STAT3 during the multi-stepped autophagic flux. Though STAT3 activity regulates autophagy in bi-directional anti- and pro-autophagic ways, nuclear STAT3, a phosphorylated and activated form of STAT3, can activate transcription of Bcl-2 family members to inhibit autophagy [54,55]. Moreover, Beclin-1 is also a potential target of STAT3. STAT3 phosphorylation leads to the reduction of Beclin-1 mRNA by directly binding to its promoter region, and the inhibition of Beclin-1 was restored by dominant-interfering mutant of STAT3 [56]. However, the relevance cytoplasmic STAT3 in autophagy regulation is also important, since the most of main autophagy process happens in the cytoplasm [57]. In 2012, Shen et al. [58] reported an unintended action from a series of STAT3 inhibitors. Pharmacological and genetic suppression of STAT3 activity led to the stimulation of protein kinase R, resulting in the induction of autophagy, while overexpression of STAT3 variants inhibited it. Indeed, further investigation is required to identify and distinguish multifaceted roles of STAT3 in autophagy. However, collectively, studies suggest that the restoration of STAT3-inhibited autophagy may represent an important mechanism for cancer treatment. In line, VA treatment clearly induced the expression of autophagy-related proteins (Figure 5E). Increased transition of LC3 identified promotion in autophagy flux, which was supported by the upregulation of Beclin-1, the key player of autophagy [59], followed by increased ATG12 protein [60] and MEK/ERK, which participates in the late stage of autophagy [35]. These in vivo results, along with similar observations in the VA-treated COC vitro model, sufficiently showed the inhibition of STAT3 by VA treatment subsequently led to the induction of autophagy of B16BL6 melanoma cells.

The significant relevance between inflammation and cancer has been well-appreciated for years, but it was only until the recent 20 years when evidence begun to reveal the actual interaction that inflammation itself is essential and perhaps a cause, rather than being a consequence, of cancer [61]. Inflammation is another important factor which connects obesity, a chronic state of low-grade inflammation, and cancer. As described above, several cytokines and inflammatory factors induce phosphorylation of STAT3, which in turn increases survival and metastatic potential in melanoma. In this study, we could not observe notable regulation in inflammatory markers (JNK and COX-2) from VA treatment; however, significantly decreased phosphorylation of p38 suggested an indirect hint on the suppressive events on inflammation by VA (Figure 5C).

Since Wu et al. [62] reported beige adipocyte as a distinct type of adipocyte with the ability to dissipate heat like brown adipocytes, interest on therapeutic activation of beige adipocyte development has risen in the field of obesity. Trans-differentiated from traditional white adipocytes, beige adipocytes exert UCP1-mediated thermogenic action to consume accumulated lipid and produce heat [63]. In our results, factors from the thermogenic process, including UCP1, PGC1α, and PRDM16, were highly increased in iWAT of VA-treated mice, and their eWAT also showed a significant increase in UCP1 expression (Figure 3A and Figure 4A). Consistently, lipolysis was induced by VA treatment, as shown by increased related markers, such as HSL, ATGL, and CGI58 (Figure 3B). Moreover, AMPK, the key regulator of energy metabolism, was activated by VA administration (Figure 4C). The alteration in lipolysis and non-shivering thermogenesis led to a notable decrease in lipid droplet size within the WATs (Figure 3C and Figure 4D). These results showing that VA can increase thermogenic potential in white adipocytes fall in line with our previous study, in which we introduced the anti-obese effect of VA [24]. Based on the negative impact on melanoma aggressiveness caused from excess mass of adipose tissue, we herein conclude reduced melanoma size not only by controlling apoptosis and autophagy in cancer, but also by ameliorating obesity factors.

One concern of this study was the prevalence of cancer-associated cachexia. Cachexia is observed in 50–80% of all cancer patients [64], and it is associated with excessive body weight loss, adipose tissue, and skeletal muscle wasting, accompanied by altered inflammatory signals [65]. The most serious problems of cachexia which affect cancer patients are the negatively impacted quality of life and decreased sensitivity to anticancer therapies. In cancer patients with cachexia, defined as >5% of body weight loss in the half year, response to cancer treatment drops; consequently, affected by this and other multiple factors, overall survival is significantly decreased [66]. To date, there is no perfect cure for cancer-associated cachexia, but the best hope is to prevent its development by early intervention [67]. Intercommunication between cancer and adipose tissues involves UCP1-regulated energy expenditure, which leads to fat wasting in cachexia, and, in this context, VA treatment has induced UCP1 activity. However, our results point out that this effect of VA is promising and still beneficial. VA treatment did not cause serious illness to mice besides lipid reduction. Although WAT weights were decreased by melanoma injection itself, we believe that mice were not cachectic, as B16BL6-injected mice did not show any muscle loss nor VA-induced muscle atrophy factors such as MuRF1 or MAFbx (Figure 2). In terms of preventing refractory cachexia before cancer pathologically impacts the whole-body metabolism, VA treatment showed promising effects by significantly reducing the size of the melanoma tissue. We observed that VA-induced body weight loss of subjects, mostly affecting lipid-accumulating organs/tissues such as WAT and liver, without affecting muscle tissue mass or increasing atrophy-related factors; thus, it allowed us to understand that VA did not drive a cachectic reaction whatsoever. Moreover, based on the fact that STAT3 is one of the responsible mechanisms of cancer-associated cachexia [68], our results, which have shown suppressed STAT3 activation by VA, offer another reason to support our conclusion.

Fueled by the growth of the global economy, changes in lifestyle and diet highly impacted the epidemiology of obesity. Prevalence of obesity is growing rapidly, even to a two- or three-times higher rate over the recent decades [69]. According to this epidemiology, aside from the negative impact of obesity on chronic diseases and overall health, it is now inevitable to understand a considerable portion of diseases, such as cancer, in a comorbidity situation accompanied by obesity. In this notion, we have conducted a comorbidity model by feeding C57BL6 mice with an HFD, followed by an allograft of melanoma, and investigated the effect of VA in regards of STAT3-related mechanisms. We observed beneficial effects of VA on both diseases, by reducing WAT accumulation and suppressing cancer growth. These effects were due to a significant repress in STAT3 activity, subsequently resulting in induced apoptotic and autophagic cell death events in the melanoma tissue, whereas VA increased non-shivering thermogenesis in WATs.

## 5. Conclusions

In this study, we investigated the beneficial effect of VA in a newly established COC model of HFD and B16BL6 melanoma cell injection. VA ameliorated obesity parameters, such as body weight gain, WAT weight, and lipid droplet size, by inducing browning and lipolysis in iWAT and AMPK activation in eWAT. Furthermore, growth of allografted B16BL6 melanoma was also suppressed by VA treatment. This was due to increased apoptosis and autophagy signaling pathway as a result of VA-inhibited STAT3 phosphorylation. Results from an in vitro COC model of adipocyte CM-treated B16BL6 cells confirmed the effect of VA on STAT3 and autophagy. In short, based on these findings, we suggest VA as a promising therapeutic agent for comorbidity of cancer and obesity.

## Supplementary Materials

Supplementary materials can be found at https://www.mdpi.com/2218-273X/10/8/1098/s1. Figure S1. Effect of VA on lipolysis markers in liver of COC mice.
