# Supplementary material for: Vanillic Acid Improves Comorbidity of Cancer and Obesity through STAT3 Regulation in High-Fat-Diet-Induced Obese and B16BL6 Melanoma-Injected Mice
Source: Biomolecules. 2020 Jul 24;10(8):1098. doi: 10.3390/biom10081098 (PMC7464557; doi:10.3390/biom10081098)
Supplement: Supplementary file 1 [file biomolecules-10-01098-s001.pdf]

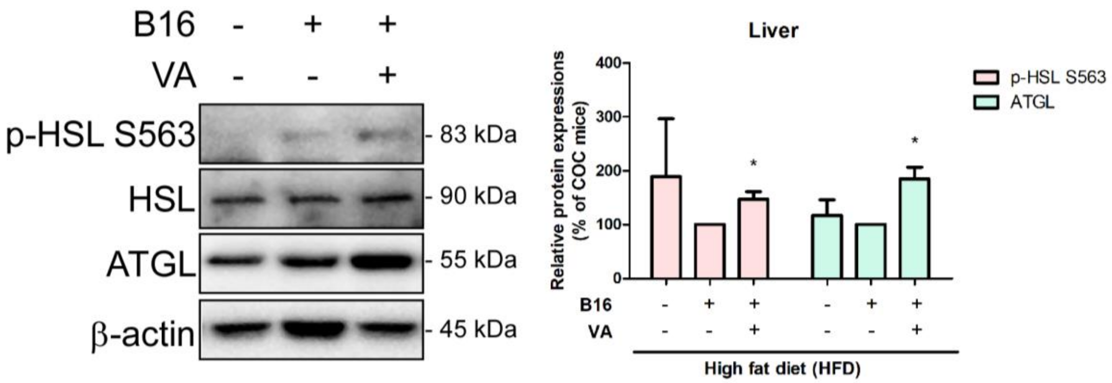


**Supplementary Figure S1.** Effect of VA on lipolysis markers in liver of COC mice. (A) Protein levels of p-HSL and ATGL were analyzed by a Western blot analysis. p-HSL was expressed relative to total HSL and ATGL was expressed relative to β-actin. All data are expressed as mean ± S.E.M. ($n = 3$). $^{*}p < 0.05$ vs. vehicle-treated COC mice. COC, cancer-obesity comorbidity; HFD, high fat diet; VA, vanillic acid.
